# Absence of GluN2A in hippocampal CA1 neurons leads to altered dendritic structure and reduced frequency of miniature excitatory synaptic events

**DOI:** 10.1093/braincomms/fcaf124

**Published:** 2025-03-26

**Authors:** Farhana Yasmin, Katie F M Marwick, Daniel W Hunter, Sarfaraz Nawaz, Grant F Marshall, Sam A Booker, Giles E Hardingham, Peter C Kind, David J A Wyllie

**Affiliations:** Centre for Discovery Brain Sciences, University of Edinburgh, Hugh Robson Building, Edinburgh EH8 9XD, UK; Simons Initiative for the Developing Brain, University of Edinburgh, Hugh Robson Building, Edinburgh EH8 9XD, UK; Centre for Discovery Brain Sciences, University of Edinburgh, Hugh Robson Building, Edinburgh EH8 9XD, UK; Simons Initiative for the Developing Brain, University of Edinburgh, Hugh Robson Building, Edinburgh EH8 9XD, UK; Centre for Discovery Brain Sciences, University of Edinburgh, Hugh Robson Building, Edinburgh EH8 9XD, UK; Simons Initiative for the Developing Brain, University of Edinburgh, Hugh Robson Building, Edinburgh EH8 9XD, UK; Centre for Discovery Brain Sciences, University of Edinburgh, Hugh Robson Building, Edinburgh EH8 9XD, UK; Simons Initiative for the Developing Brain, University of Edinburgh, Hugh Robson Building, Edinburgh EH8 9XD, UK; Centre for Discovery Brain Sciences, University of Edinburgh, Hugh Robson Building, Edinburgh EH8 9XD, UK; Simons Initiative for the Developing Brain, University of Edinburgh, Hugh Robson Building, Edinburgh EH8 9XD, UK; Centre for Discovery Brain Sciences, University of Edinburgh, Hugh Robson Building, Edinburgh EH8 9XD, UK; Simons Initiative for the Developing Brain, University of Edinburgh, Hugh Robson Building, Edinburgh EH8 9XD, UK; Centre for Discovery Brain Sciences, University of Edinburgh, Hugh Robson Building, Edinburgh EH8 9XD, UK; Simons Initiative for the Developing Brain, University of Edinburgh, Hugh Robson Building, Edinburgh EH8 9XD, UK; United Kingdom Dementia Research Institute at The University of Edinburgh, Edinburgh Medical School, Edinburgh EH16 4SB, UK; Centre for Discovery Brain Sciences, University of Edinburgh, Hugh Robson Building, Edinburgh EH8 9XD, UK; Simons Initiative for the Developing Brain, University of Edinburgh, Hugh Robson Building, Edinburgh EH8 9XD, UK; Centre for Discovery Brain Sciences, University of Edinburgh, Hugh Robson Building, Edinburgh EH8 9XD, UK; Simons Initiative for the Developing Brain, University of Edinburgh, Hugh Robson Building, Edinburgh EH8 9XD, UK

**Keywords:** *Grin2a*, GluN2A, NMDA receptor, CA1, hippocampus

## Abstract

GluN2A is a NMDA receptor subunit postulated as important for learning and memory. In humans, heterozygous loss of function variants in the gene encoding it (*GRIN2A*) increase the risk of epilepsy, intellectual disability and schizophrenia. Haploinsufficient mouse models show electrophysiological abnormalities and thus to improve and widen understanding of the pathogenesis of *GRIN2A*-associated disorders in humans, this study aimed to assess the impact of *Grin2a* absence and haploinsufficiency on core neuronal and synaptic properties in genetically modified rats. Electrophysiological whole-cell current- and voltage-clamp recordings were made from CA1 pyramidal neurons in acute hippocampal slices from wild-type and *Grin2a* heterozygous (*Grin2a^+/−^*) and homozygous (*Grin2a^−/−^*) knock out rats aged postnatal day 27–34. While reduced levels or absence of GluN2A did not affect neuronal excitability or intrinsic membrane properties in both *Grin2a^+/−^* and *Grin2a^−/−^* rats, we found a reduced frequency of miniature excitatory post synaptic currents and a reduced density of proximal dendrites suggestive of a reduced number of excitatory synapses. Recordings from CA1 neurons in slices prepared from *Grin2a^+/−^* and *Grin2a^−/−^* rats revealed there was a reduced ratio of the current mediated by NMDA receptors compared to AMPA receptors, while in *Grin2a^−/−^* recordings, there was a slowing of the decay time-constant of the NMDA receptor-mediated excitatory postsynaptic currents. Moreover, neither summation of sub-threshold excitatory postsynaptic potentials nor summation of supra-threshold excitatory postsynaptic potentials to initiate action potential firing in CA1 pyramidal neurons indicated any dependence on GluN2A. We conclude that reduced levels of GluN2A alters the kinetics of NMDA receptor-mediated synaptic events and dendritic structure of CA1 neurons, but do not affect several other core neuronal functions. These relatively subtle changes are consistent with the largely intact neural functioning of the majority of humans carrying *GRIN2A* loss of function variants. Further research could explore whether the changes in synaptic properties we observed contribute to alterations in higher level circuit dynamics and computation, which may manifest as disorders of cognition and excitability in humans.

## Teaser

Yasmin, Marwick *et al*. report that GluN2A *N*-methyl-D-aspartate receptor subunit absence in CA1 pyramidal neurons leads to a decrease in the frequency of excitatory miniature synaptic currents and decreased density of proximal dendrites. Nevertheless, loss of this *N*-methyl-D-aspartate receptor subunit does not impact excitatory postsynaptic potential summation or synaptically-evoked action potential firing.

## Graphical Abstract

**Graphical Abstract:**
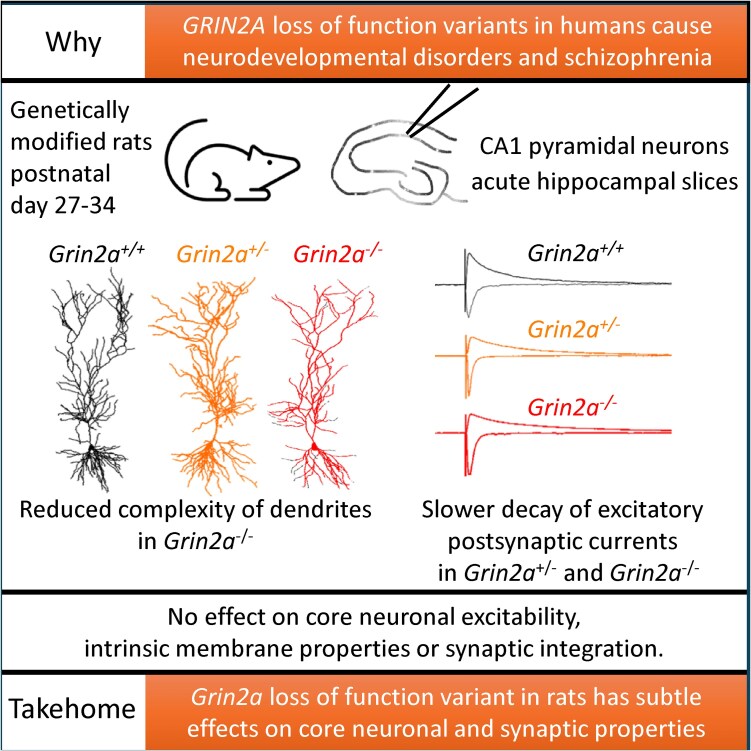

## Introduction

NMDA receptors are a cation-permeable, voltage-dependent, glutamate-gated ion channel receptors that act as molecular coincidence detectors due to their blockade by Mg^2+^ ions at hyperpolarized membrane potentials.^1,2^ This unusual set of properties makes NMDA receptors well suited to transmute patterns of synaptic input into modulation of synaptic strength, the cellular substrate of learning and memory.^3^ More recently there has been a recognition that genetic variations leading to either loss or gain of function of NMDA receptors are linked to diverse neuropsychiatric disorders.^4,5^

NMDA receptors assemble as heterotetramers, comprising two obligatory GluN1 subunits and two other subunits, of which GluN2A and GluN2B predominate in glutamatergic principal neurons of the mammalian forebrain,^6-8^ and which are encoded by *GRIN2A* and *GRIN2B,* respectively. The identity of the GluN2 subunit influences receptor properties that influence synaptic plasticity, Mg^2+^ sensitivity, Ca^2+^ permeability and channel open probability.^9-11^ GluN2 subunit levels are developmentally regulated: early in rodent forebrain development GluN2A is much less abundant than GluN2B but increases over development, reducing the GluN2B:GluN2A ratio over time.^6-8^ In humans, a similar increase in GluN2A levels during development is observed.^12^ Thus, in the forebrain, GluN1/GluN2B diheteromers predominate prenatally and early in development, but postnatally GluN1/GluN2A/GluN2B triheteromers become the dominant synaptic NMDA receptor.^13^

Over the last decade, genetic studies have reported a range of ultra-rare variants in GluN NMDA receptor subunits^4,5^ with these variants being present almost exclusively heterozygously and associated with a diversity of neurodevelopmental conditions, chiefly intellectual disability and epilepsy, with onset predominantly in childhood.^4^ Over 300 variants have been identified in *GRIN2A* and approximately one third of these are null variants resulting in absent protein.^14,15^ Null variant carriers have a milder phenotype than most gain-of-function variants, typically have no or mild intellectual disability, focal (not generalised) seizures and speech and language delay,^14^ and the clinical impact can be sufficiently mild to allow carriers to reproduce.^4^

*GRIN2A* has also been identified as a converging site of common and rare genetic variation in neuropsychiatric disorders presenting in adolescence and adulthood, particularly schizophrenia. The largest genome-wide association study in schizophrenia to date reported a pathogenic role for common variants in *GRIN2A* and highlighted the gene as a strong candidate for further functional studies.^16^ Although the mechanism by which these variants influence risk for schizophrenia is unknown, the intronic location of 62 out of 63 *GRIN2A* fine-mapped single nucleotide polymorphisms implicates changes in expression or alternative splicing rather than gain of function events, and single-cell RNA sequencing of post-mortem samples shows reductions in multiple excitatory cell types in schizophrenia.^17^ Rare variants in *GRIN2A* are also associated with schizophrenia; with heterozygous protein-truncating *GRIN2A* variants conferring a very high odds ratio for schizophrenia of 18 (95% confidence interval: 3.74–172).^12^

Based on this robust genetic evidence, understanding the mechanistic consequences of *GRIN2A* haploinsufficiency is a key next step in understanding the pathogenesis of multiple important human neurodevelopmental disorders. Rodent models provide a tractable route to do this. A *Grin2a* knock-out mouse model has been available for some time, which has been found to be generally healthy and viable but with alterations in synaptic plasticity and memory^18-20^ and in interneuron maturation.^15^ Recently, studies have begun to report on mouse models of *Grin2a* haploinsufficiency identifying transient changes in hippocampal volume at P30^21^, large shifts in gene expression in the hippocampus (and other brain regions) at 4 weeks and older,^22^ delays in interneuron maturation,^15^ and changes to brain activity during sleep, with both increases in gamma power^22,23^ and more frequent sleep spindles observed.^23^ In parallel, we have developed rat models in which GluN2A is reduced or completely absent^14,24^ not least because of the importance of validating physiological and behavioural phenotypes across preclinical models but because of the potential for task-free functional imaging to be performed in the rat brain.^25^

Strong genetic evidence thus now highlights reduced levels of GluN2A as contributing to the pathophysiology of human disorders of cognition and excitability. In the present study, we conducted an investigation into the impact of *Grin2a* haploinsufficiency and complete absence on basic neuronal properties and synaptic functioning in two *Grin2a* rat models. We found that hippocampal neurons in acute brain slices from *Grin2a^+/−^* and *Grin2a^−/−^* rats showed no significant alteration in intrinsic properties. The chief difference was a reduction in frequency but not amplitude of miniature excitatory postsynaptic currents (EPSCs), associated with a reduced complexity of proximal dendrites. These relatively subtle changes are consistent with the largely intact neural functioning of the majority of humans carrying *GRIN2A* loss of function variants.

## Materials and methods

### Animals

Two independently-generated Long-Evans Hooded rat lines were used in this study, both with modifications to exon 11 of the *Grin2a* gene resulting in a non-translated gene product. Line 1 rats had exon 11 deleted. Line 2 rats had exon 11 flanked by LoxP sites, and an unintended single base pair deletion early in exon 11, resulting in a frameshift and early termination of transcript (c.1783delC, p.H595Tfs*57). The LoxP sites were thus redundant. These rats were generated by pronuclear microinjection of mRNA encoding the enzyme Cas9, single guide RNA binding to 5′ and 3′ of exon 11 of *Grin2a*, and a pUC57 plasmid containing 2142 bp of donor DNA (containing exon 11 flanked by LoxP sites) into single-cell Long-Evans Hooded rat embryos before being implanted into pseudo-pregnant mothers. The resulting live births were screened by PCR for either insertion of donor DNA via homology directed repair or genomic deletions due to repair by non-homologous end joining of double stranded breaks targeted to either side of exon 11. Rats where non-homologous end joining had occurred showed a large deletion (1065 base pairs) spanning exon 11 and are referred to here as Line 1.^14,24^ Rats where the two LoxP sites had been incorporated were then modified further by a second round of Crispr/Cas9 injections, this time with a 159 bp donor oligonucleotide containing five point mutations in exon 11. This second round of modification introduced the intended point mutations but also a one base pair deletion to 5′ of the point mutations (c.1783delC), effectively knocking out the gene. These rats are referred to here as Line 2. The single guide RNA design and generation of the founder animals was performed by Horizon Discovery Group plc (St. Louis, MO, USA). Confirmation by Sanger sequencing was performed by PCR amplification of a genomic region spanning the donor DNA section and including endogenous DNA to 5′ and 3′.

Animals heterozygous for the mutations were then crossed to give offspring that were either wild-type (*Grin2a^+/+^*), heterozygous (*Grin2a^+/−^*) or homozygous (*Grin2a^−/−^*). Offspring, both male and female, from these litters were culled for tissue between postnatal days 27 and 36 (P27–36). Rats were housed under a standard 12:12 h light/dark cycle and received food and water *ad libitum.* Experiments performed in this study were in accordance with the United Kingdom Animals (Scientific Procedures) Act of 1986 under the Home Office licence P1351480E.

### Acute hippocampal slice preparation

Animals were decapitated under isoflurane anaesthesia, and their brains quickly removed. Four hundred micrometre horizontal hippocampal slices were prepared using a vibratome (Leica VT 1200S). Slices were collected in carbogenated (95% oxygen, 5% CO_2_) ice-cold dissection buffer containing the following (in mM): 86 NaCl, 1.2 NaH_2_PO_4_, 2.5 KCl, 25 NaHCO_3_, 25 glucose, 75 sucrose, 0.5 CaCl_2_ and 7 MgCl_2_. Slices were incubated for 30 min at 30°C in artificial cerebrospinal fluid containing the following (in mM): 124 NaCl, 1.2 NaH_2_PO_4_, 2.5 KCl, 25 NaHCO_3_, 20 glucose, 2 CaCl_2_ and 1 MgCl_2_, bubbled with 95% oxygen and 5% CO_2_.

### Whole-cell patch-clamp recordings

For electrophysiological recordings, slices were placed in a submersion chamber heated to 32 ± 1°C with an inline heater (LinLab Scientifica, UK) and perfused with carbogenated artificial cerebrospinal fluid at a rate of 6–8 mL/min. Slices were visualized with an upright microscope (ZEISS Axioskop 50, Oberkochen, Germany). Whole-cell recording electrodes were pulled using borosilicate glass capillaries (1.5 mm outer, 0.7 mm inner diameter) on a horizontal puller (P-97, Sutter Instrument Co., Novato, CA, USA). For current-clamp recordings, pipettes were filled with a potassium gluconate-based solution (in mM: 120 potassium gluconate, 20 KCl, 4 NaCl, 0.1 EGTA, 10 HEPES, 4 Na_2_-ATP, 0.3 Na_2_-GTP, 10 Na_2_-phosphocreatinine, pH 7.3, 290–310 mosmol l^−1^), with a final resistance of 3–5 MΩ. Recordings were performed using a Multiclamp 700B amplifier (Molecular Devices, Sunnyvale, CA, USA). Intrinsic physiology of neurons was recorded in current-clamp mode with a family of current pulses (−100 to 400 pA, 25 pA steps, 500 ms duration, 3 repetitions) and input resistance measured using small hyperpolarizing steps (−10 pA, 500 ms duration, 30 repetitions). Voltage and current signals were low-pass filtered at 10 and 2 kHz, respectively, using the built in Bessel filter of the amplifier, digitized at 20 kHz (Digidata 1440, Molecular Devices) and acquired with pCLAMP software (pClamp 10, Molecular Devices). Data were analysed offline using open-source Stimfit software (Line 1) or a custom-made MATLAB script (Line 2; https://github.com/adj-4/IntrinsicAnalysis/tree/main/IntrinsicAnalysis/code).

For voltage-clamp recordings of evoked and spontaneous glutamatergic synaptic currents, a caesium gluconate-based internal solution (110 Cs-gluconate, 20 CsCl, 4 NaCl, 0.2 EGTA, 10 HEPES, 4 Mg-ATP, 0.3 Na_2_-GTP, 10 Na_2_-phosphocreatinine, 5 QX-314; pH 7.35, 290–310 mosmol l^−1^) was used with the GABA_A_ receptor antagonists, picrotoxin (50 μM) or gabazine (5 μM) added to the external recording solution. Miniature EPSCs were recorded using the same protocols as spontaneous EPSCs, except that the external solution also contained 300 nM tetrodotoxin (TTX). Biotinylated-lysine (0.1%; Biocytin, Invitrogen, UK) was included in recordings where dendritic reconstructions were required. Recordings of spontaneous inhibitory postsynaptic currents (IPSCs) and miniature IPSCs, at −70 mV, were made using a high Cl^−^-based internal solution (140 CsCl, 10 HEPES, 5 Mg-ATP, 1 Na_2_-GTP, 2 MgCl_2_, 0.5 EGTA, 10 Na_2_-phosphocreatine, 5 QX-314; pH 7.35, 290–310 mosmol l^−1^) with 6-cyano-7-nitroquinoxaline-2,3-dione (CNQX; 10 μM) added to the external recording solution [and TTX (300 nM) added for miniature IPSC recordings]. Continuous current traces of 5-min duration (recorded at least 5 min after achieving whole-cell configuration) were analyzed using the Mini Analysis Program (Synaptosoft, Fort Lee, NJ, USA) with a threshold for detection set at ≥ 6 pA.

For NMDA:AMPA ratios, area CA3 was removed from the slice before transfer to a submerged chamber. Evoked EPSCs were elicited via extracellular stimulation with a bipolar electrode, connected to a stimulus isolator, placed in Schaffer collateral pathway. Traces were collected every 20 s. Voltage-clamp recordings, in the presence of GABA_A_ receptor antagonists, picrotoxin (50 μM) or gabazine (5 μM) were made at −70 mV to isolate AMPA currents. CNQX (10 μM, used for recordings from Line 1) or 2,3-dioxo-6-nitro-7-sulfamoyl-benzo[f]quinoxaline (NBQX; 10 μM, used for recordings from Line 2) was added to the bath to block AMPA receptor-mediated currents and reveal NMDA receptor-mediated only currents at +40 mV.

Sub-threshold summation of excitatory postsynaptic potentials (EPSPs) was measured with trains of 5 stimuli delivered at 10, 20 and 50 Hz (10 s inter-sweep interval); 20 traces were collected for each frequency tested. Stimulus intensity was adjusted to elicit a first EPSP of approximately 1–3 mV in amplitude and was kept constant throughout the stimulus trains. For these experiments to avoid initiation of action potential firings QX-314 (5 µM) was included in the pipette-filling solution. For supra-threshold summation of EPSPs to evoke action potential firing, the stimulus intensity was adjusted to elicit a first EPSP of approximately 5–8 mV in amplitude and was then kept constant for all subsequent trains.

Series resistance (*R*_S_) was constantly monitored throughout the recordings but was not compensated. Recordings were abandoned if initial *R*_S_ exceeded 30 MΩ or changed by more than 20% over the time course of the recording, if I_hold_ was >150 pA in voltage-clamp recordings or membrane potential was more depolarized than −50 mV in current-clamp recordings.

### Synaptosome preparation

Male rats were anaesthetized with isoflurane and decapitated. The hippocampi were dissected in ice cold sucrose-EDTA buffer (in mM: 320 sucrose, 1 EDTA, 5 Tris, pH 7.4). The tissue was snap-frozen and stored at −80°C until synaptosome preparation. On the day of preparation, the discontinuous Percoll-density gradient with 1X sucrose-EDTA (3% uppermost, 10% middle and 23% bottom) was made prior to homogenization. The tissue was immediately thawed at 37°C and homogenized in ice-cold sucrose-EDTA buffer in a pre-chilled Teflon glass using 5–6 ups-and-down strokes with a motorized homogenizer.^26^ The homogenates were centrifuged at 2800 rpm for 10 min. The supernatant was collected and poured very gently on the top of 3% Percoll-sucrose (Percoll, P1644, Sigma-Aldrich, UK) followed by centrifugation at 20 000 rpm for 8 min. The fraction between 23% and 10% was collected and re-suspended in HEPES-Buffered-Krebs (HBK; in mM: 118.5 NaCl, 4.7 KCl, 1.18 MgSO_4_, 10 Glucose, 1 Na_2_HPO_4_, 20 HEPES, pH 7.4 balanced with Trizma) and centrifuged at 13 000 rpm for 15 min. The pure synaptosome pellet was dissolved in RIPA buffer added with phosphatase and protease inhibitor cocktails (phosphatase inhibitor; cocktail II P5726, Cocktail III P0044, Sigma-Aldrich, UK and protease inhibitor; Roche complete mini EDTA-free protease inhibitor cocktail 4693159001, Sigma-Aldrich, UK). The sample’s temperature was maintained 4°C throughout the experiment. Proteins were estimated with MicroBCA Assay kit (Pierce BCA protein estimation kit, 23225, ThermoFisher Scientific).

### Western blots

Approximately 10 μg of the protein was separated on a precast gradient gel (4–15% Mini-PROTEAN TGX Gel, 4561086, Bio-Rad) and transferred to nitrocellulose membrane (AmershamTM Protran® Western Blotting Membrane, Nitrocellulose, GE10600002, Sigma-Aldrich) in a Bio-Rad apparatus. Membranes were incubated with 1:1 TBS1X: Odyssey Blocking Buffer (P/N-927-50003, *LI-COR* Biotech.) for an hour at RT, followed by incubation with primary antibodies (NMDAR1-1:1000, #700685, Thermo Fisher; NMDAR2A-1: 1000, #ab169873, Abcam; NMDAR2B− 1:1000, #610417, BD Biosciences, β-Actin- 1: 5000, A2228, Sigma-Aldrich) at 4°C overnight. Membranes were washed with 1X TBST (0.1% Tween 20) and incubated with secondary antibodies (IRDye 800CW Goat anti Rabbit IgG-1: 10,000, P/N 925-32211, IRDye 680LT Goat anti Mouse IgG P/N 925-68020, LI-COR Biotechnology) for an hour at RT. After washing with 1X TBST, membranes were dried and digitally scanned using the Odyssey CLx Imaging System, *LI-COR*, UK Ltd. The density of individual bands was measured by Odyssey software, *LI-COR* Image Studio Lite (*LI-COR* Biosciences). Data were normalized to the respective β− actin and then normalized to wild-type (*Grin2a^+/+^*).

### Dendritic analysis

Morphological analysis of the recorded CA1 pyramidal neurons was performed as previously described.^27^ Following whole-cell recording, outside-out patches were formed and then slices fixed immediately with 4% paraformaldehyde in 0.1 M phosphate buffer, overnight at 4°C. Slices were then rinsed repeatedly in phosphate buffered saline (0.1 M phosphate buffer + 0.9% NaCl; PBS) and then incubated with Streptavidin conjugated to AlexaFluor 633 (1:500, Invitrogen, Dunfermline, UK) diluted in PBS containing 0.5% Triton-X100 and 0.05% NaN_3_, overnight at 4°C. Slices were then rinsed in PBS then phosphate buffer and mounted on glass slides with fluorescence-protecting mounting medium (Fluoromount-G, Southern Biotech, AL, USA). Recorded neurons were visualized on a laser scanning confocal microscope (SP8, Leica, Germany) with either 20× (N.A 0.75) or oil-immersion 63× (N.A 1.3) objective lenses for reconstruction. CA1 pyramidal neurons were 3-dimensionally reconstructed by combining multiple image z-stacks (1 μm steps) using the Simple Neurite Tracer plug-in for FIJI.^28^ Sholl analysis was performed on the 3-dimensionally segmented neurons, with a vector drawn from the centre of the soma to the greatest dendritic extent. Based on this distance, concentric shells (10 μm radius increments) were drawn from the origin (soma) and the number of dendritic intersections measured for each increment. For spine density analysis, high-resolution *z*-stacks (0.13 μm step size, 2.5× zoom) were imaged from secondary basal, apical oblique or apical tuft dendrites, at least 50 μm from the somata. These images were deconvolved (Huygens, Scientific Volume Imaging, the Netherlands), then spines counted manually in deconvolved images. Spine density is conveyed as the number of spines for a given length of dendrite measured.

### Statistical analysis

Values are reported as mean ± SD. Statistical comparisons were made using ‘*N*’ as number of animals. Data were averaged for each animal used, thereby reducing the risk of type-1 statistical errors resulting from pseudo-replication of the data. Average data throughout are presented as the average of each animal or group of animals. Multiple group comparisons were carried out using ANOVA followed by *post hoc* pairwise tests, performed if the *F* test was significant for a main effect. Correction for multiple comparisons is made using Holm–Sidak method. *P* < 0.05 was considered statistically significant (∗*P* < 0.05, ∗∗*P* < 0.01 and ∗∗∗*P* < 0.001). Data were analysed using Prism (GraphPad Software, La Jolla, CA, USA).

## Results

### Reduced GluN2A levels in *Grin2a*^+/−^ and *Grin2a*^−/−^ rats

**Figure 1:**
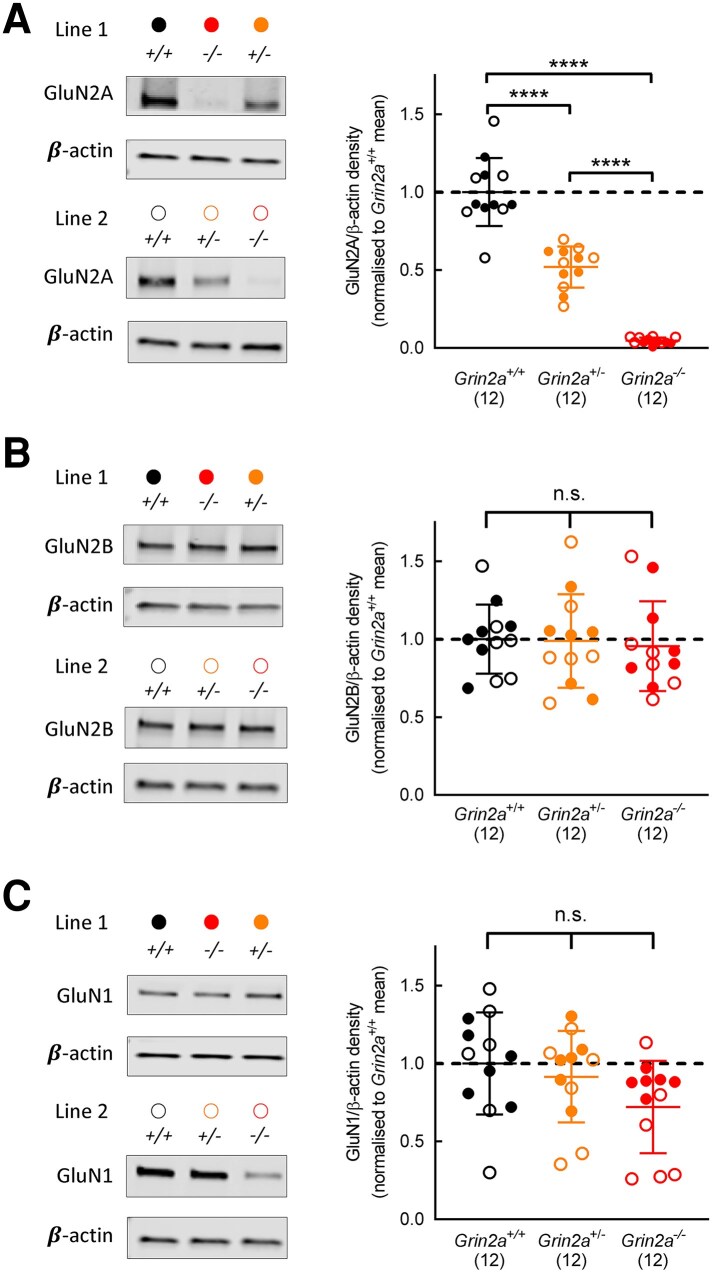
**Reduced and loss of GluN2A in two models of *Grin2a* haploinsufficiency**. (**A**) Example of western blots of GluN2A protein levels in hippocampal synaptosomes prepared from *Grin2a*^+/+^, *Grin2a*^+/−^ and *Grin2a*^−/−^ rats. In each of Line 1 and Line 2, there was approximately 50% reduction in GluN2A in synaptosomes prepared from *Grin2^+^*^/−^ rats and near complete loss of GluN2A in synaptosomes prepared from *Grin2a*^−/−^ rats [one-way ANOVA, *F*(2,33) = 124.8, *P* < 0.0001]. Normalized means for each genotype: *Grin2a^+/+^*: 1.000 ± 0.2182, N = 12; *Grin2a^+/−^*: 0.5194 ± 0.1323, N = 12; *Grin2a^−/−^*: 0.04659 ± 0.02031, N = 12. Holm-Sidak post-tests: *Grin2a^+/+^* versus *Grin2a*^+/−^, t = 7.965, *P* < 0.0001; *Grin2a^+/+^* versus *Grin2a*^−/−^, *t* = 15.8, *P* < 0.0001. (**B**) Example of western blots of GluN2B protein levels in hippocampal synaptosomes prepared from *Grin2a*^+/+^, *Grin2a*^+/−^ and *Grin2a*^−/−^ rats. In each of Line 1 and Line 2, there was no significant difference in GluN2B within each of the genotypes [one-way ANOVA, *F*(2,33) = 0.08794, *P* = 0.9160]. Normalized means for each genotype: *Grin2a^+/+^*: 1.000 ± 0.2215, N = 12; *Grin2a^+/−^*: 0.989 ± 0.3004, *N* = 12; *Grin2a^−/−^*: 0.9553 ± 0.2877, *N* = 12. (**C**) Example of western blots of GluN1 protein levels in hippocampal synaptosomes prepared from *Grin2a*^+/+^, *Grin2a*^+/−^ and *Grin2a*^−/−^ rats. In each of Line 1 and Line 2, there was no significant difference in GluN1 within each of the genotypes [one-way ANOVA, *F*(2,33) = 2.624, *P* = 0.0876]. Normalized means for each genotype: *Grin2a^+/+^*: 1.000 ± 0.327, *N* = 12; *Grin2a^+/−^*: 0.9149 ± 0.2937, *N* = 12; *Grin2a^−/−^*: 0.7207 ± 0.2965, *N* = 12. *N* numbers represent the number of animals from which homogenates were prepared (i.e. the independent biological replicate). Full western blots for Lines 1 and 2 are available as Supplemental Figs 1 and 2, respectively. Both male and female rats were used in western blots. Line 1: *Grin2a^+/+^*: 4 males, 2 females; *Grin2a^+/−^*: 4 males, 2 females; *Grin2a^−/−^*: 4 males, 2 females. Line 2: *Grin2a^+/+^*: 6 males; *Grin2a^+/−^*: 6 males; *Grin2a^−/−^*: 6 males.

To confirm that the two independent *Grin2a* lines used in this study show altered GluN2A protein levels, we measured GluN2A abundance in hippocampal synaptosome fractions from P27- P34 rats using an N-terminus antibody to GluN2A. The N-terminus antibody was chosen to detect any truncated protein products that might result from the genetic modification. As expected, GluN2A levels were reduced to approximately 50%, as is to be expected, in *Grin2a*^+/−^ rats while we considered levels of GluN2A in *Grin2a*^−/−^ rats to be negligible, and both lines were considered to be full ‘knock-outs’ for GluN2A [Fig. 1A; one-way ANOVA, *F*(2,33) = 124.8, *P* < 0.0001]. We did not detect any lower molecular weight bands in the gels and therefore conclude that no truncated protein is present in our synaptosome preparations. Levels of GluN2B (Fig. 1B) and GluN1 (Fig. 1C) in hippocampal synaptosomes prepared from *Grin2a*^+/+^, *Grin2a*^+/−^ and *Grin2a*^−/−^ rats were not significantly different, and we concluded that reduced or absent GluN2A does not lead to a detectable compensatory increase in the levels of these other NMDA receptor subunits [GluN2B: one-way ANOVA, *F*(2,33) = 0.08794, *P* = 0.9160; GluN1: one-way ANOVA, *F*(2,33) = 2.624, *P* = 0.0876].

### No changes in intrinsic excitability or membrane properties with reduced GluN2A levels

**Figure 2:**
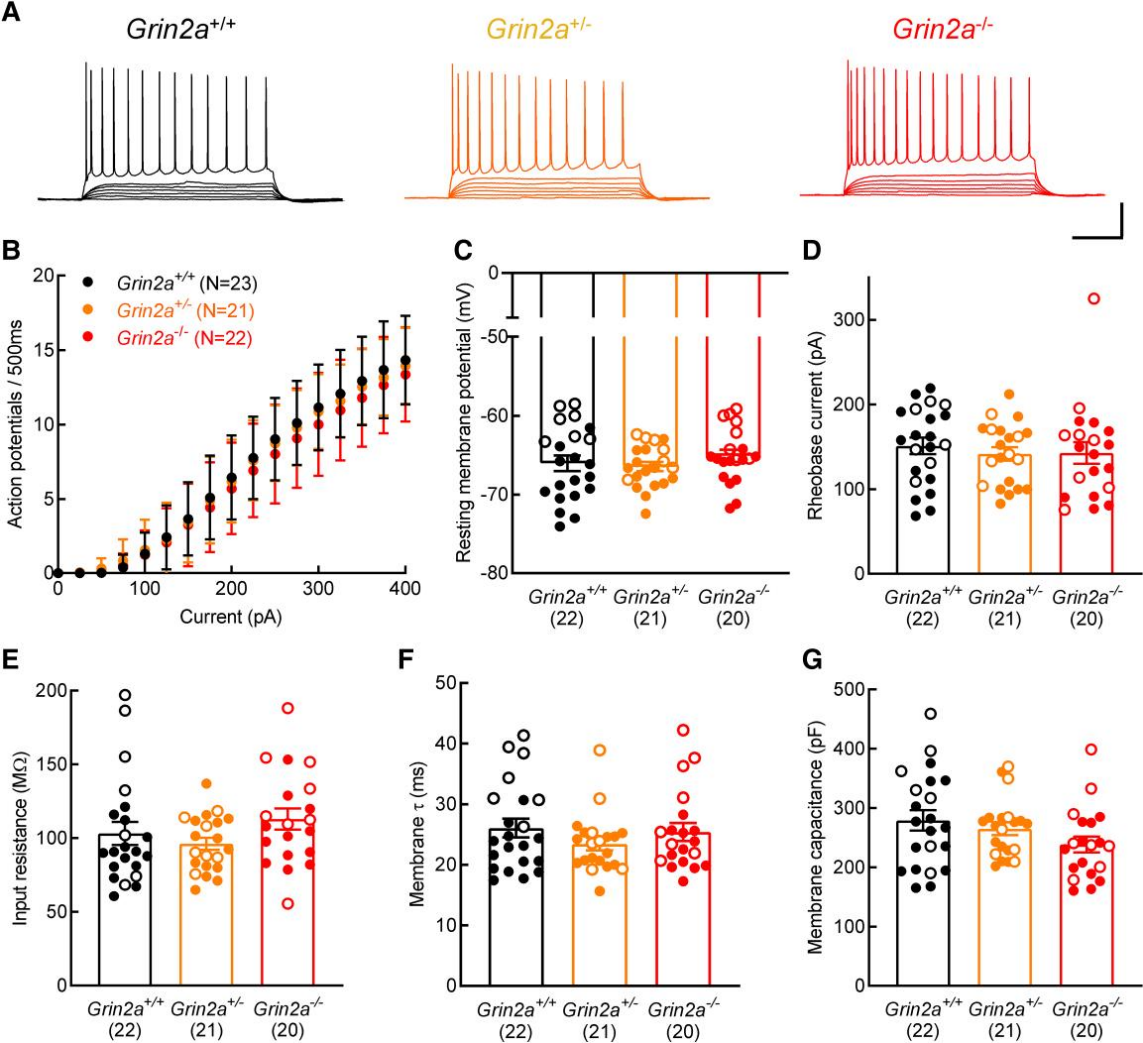
**Reduced levels and loss of GluN2A does not alter intrinsic excitability and membrane properties of CA1 pyramidal neurons.** (**A**) Representative current-clamp traces showing action potential firing in response to depolarizing current injections (500 ms; + 25 pA steps) from either *Grin2a^+/+^* (left traces), *Grin2a^+/−^* (middle traces) or *Grin2a^−/−^* (right traces) CA1 pyramidal neurons. (**B**) There is no change in the number of action potentials discharged for equivalent depolarizing current injections for recordings made from CA1 pyramidal neurons in each of the three genotypes [two-way ANOVA, effect of genotype: *F*(2,63) = 0.5449, *P* = 0.5826]. (**C**) Resting membrane potentials are unaltered in *Grin2a*^+/+^, *Grin2a*^+/−^ and *Grin2a*^−/−^ CA1 pyramidal neurons [one-way ANOVA, *F*(2,60) = 0.734, *P* = 0.4844. Mean values for each genotype: *Grin2a^+/+^*: −66.04 ± 4.68 mV, *Grin2a^+/−^*: −66.46 ± 2.67 mV, *Grin2a^−/−^*: −65.08 ± 3.48 mV]. (**D**) No alteration in the mean rheobase current required to initiate action potential firing [one-way ANOVA, *F*(2,60) = 0.258, *P* = 0.7738. Mean values: *Grin2a^+/+^*: 151.40 ± 45.86 pA, *Grin2a^+/−^*: 142.00 ± 36.61 pA, *Grin2a^−/−^*: 143 ± 57.06 pA]. (**E**) Input resistances are unaltered in *Grin2a*^+/+^, *Grin2a*^+/−^ and *Grin2a*^−/−^ CA1 pyramidal neurons [one-way ANOVA, *F*(2,60) = 1.599, *P* = 0.2107. Mean values: *Grin2a^+/+^*: 103.20 ± 36.53 MΩ, *Grin2a^+/−^* : 96.13 ± 18.90 MΩ, *Grin2a^−/−^*: 112.9 ± 31.72 MΩ]. (**F**) Membrane time-constants do not differ between genotypes [one-way ANOVA, *F*(2,60) = 0.999, *P* = 0.3741. Mean values: *Grin2a^+/^*: 26.05 ± 7.25 ms, *Grin2a^+/−^*: 23.41 ± 4.86 ms, *Grin2a^−/−^*: 25.41 ± 6.67 ms]. (**G**) The membrane capacitance of CA1 pyramidal neurons is not influenced by altered GluN2A levels [one-way ANOVA, *F*(2,60) = 2.096, *P* = 0.1319. Mean values: *Grin2a^+/+^*: 278.90 ± 80.77 pF, *Grin2a^+/−^*: 264.90 ± 49.01 pF, *Grin2a^−/−^*: 238.3 ± 59.75 pF]. (**B–G**) The N numbers, indicated in parentheses, represent the number of animals from which recordings were obtained (i.e. the independent biological replicate). Filled symbols represent data from Line 1, open symbols from Line 2. Scale bar in **A**, 25 mV, 150 ms. Both male and female rats were used to generate datasets in **B**, line 1: *Grin2a^+/+^*: 11 males, 5 females; *Grin2a^+/−^*: 10 males, 4 females; *Grin2a^−/−^*: 12 males, 2 females. Line 2: *Grin2a^+/+^*: 7 males; *Grin2a^+/−^*: 7 males; *Grin2a^−/−^*: 8 males. Both male and female rats were used to generate datasets in **C–G**, line 1: *Grin2a^+/+^*: 10 males, 5 females; *Grin2a^+/−^*: 10 males, 4 females; *Grin2a^−/−^*: 10 males, 2 females. Line 2: *Grin2a^+/+^*: 7 males; *Grin2a^+/−^*: 7 males; *Grin2a^−/−^*: 8 males.

We next investigated whether reduction or complete loss of GluN2A affected intrinsic excitability and membrane properties of CA1 pyramidal neurons. All recordings were made from P27-36 rats, at which age GluN2A levels are similar to those seen in the mature CNS.^7^ Depolarizing current injections gave rise to typical action potential firing in CA1 pyramidal neurons in slices prepared from *Grin2a*^+/−^ and *Grin2a*^−/−^ rats compared to *Grin2a*^+/+^ recordings (Fig. 2A) with no change in the number of action potentials discharged for equivalent depolarizing current injections [Fig. 2B; two-way ANOVA, effect of genotype: *F*(2,63) = 0.5449, *P* = 0.5826]. In addition, there were no differences in the resting membrane potentials, recorded in the first minute after gaining whole cell access [Fig. 2C; one-way ANOVA, *F*(2,60) = 0.734] the rheobase currents required to elicit action potential firing [Fig. 2D; one-way ANOVA, *F*(2,60) = 0.258, *P* = 0.7738] the input resistance [Fig. 2E; one-way ANOVA, *F*(2,60) = 1.599, *P* = 0.2107], membrane time-constant [Fig. 2F; ; one-way ANOVA, *F*(2,60) = 0.999, *P* = 0.3741], or membrane capacitance [Fig. 2G; one-way ANOVA, *F*(2,60) = 2.096, *P* = 0.1319] recorded from CA1 pyramidal neurons from each of the three genotypes.

### Decreased NMDAR:AMPAR ratios and slower decay of glutamatergic EPSCs in *Grin2a*^+/−^ and *Grin2a*^−/−^ CA1 pyramidal neurons

**Figure 3:**
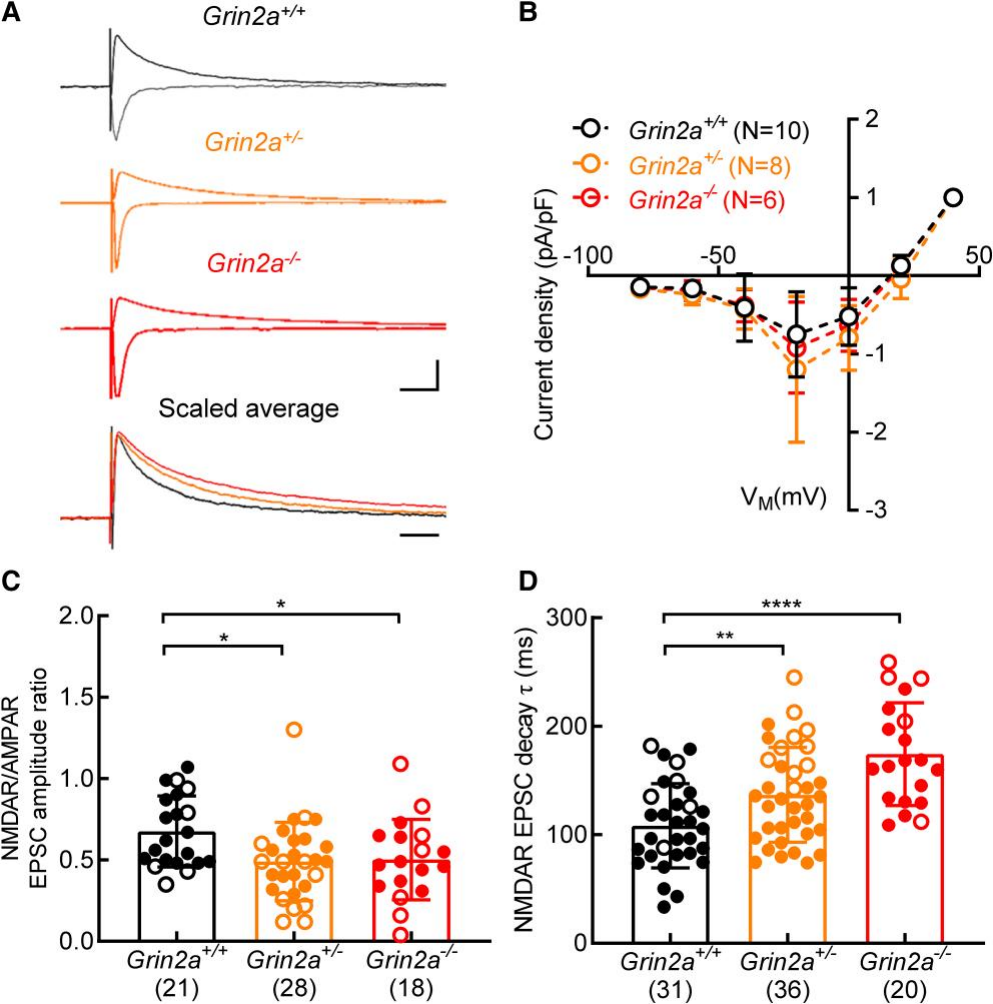
**Altered GluN2A levels change NMDA to AMPA receptor ratios and slows the decay of the NMDA receptor component of the glutamatergic EPSC in CA1 pyramidal neurons.** (**A**) Examples of glutamatergic EPSCs recorded at −70 and +40 mV from *Grin2a*^+/+^, *Grin2a*^+/−^ and *Grin2a*^−/−^ CA1 pyramidal neurons following stimulation of the Schaffer collateral/commissural pathway. The peak scaled outward EPSCs recorded at +40 mV reveal a slowing of the decay of the current in *Grin2a*^+/−^ and *Grin2a*^−/−^ neurons (quantified in **D**). (**B**) The current–voltage relationship and normalized current densities of glutamatergic EPSCs is similar for all genotypes. (**C**) Altered GluN2A levels reduces the NMDA to AMPA receptor ratio [one-way ANOVA, *F*(2,64) = 4.214, *P* = 0.0191. Mean values for each genotype: *Grin2a^+/+^*: 0.6752 ± 0.219, *N* = 21; *Grin2a^+/−^*: 0.49 ± 0.2408, *N* = 28; *Grin2a^−/−^*: 0.5028 ± 0.248, *N* = 18. Holm–Sidak post-tests: *Grin2a^+/+^* versus *Grin2a*^+/−^, *t* = 2.717, *P* = 0.0169; *Grin2a^+/+^* versus *Grin2a*^−/−^, *t* = 2.273, *P* = 0.0264]. (**D**) Loss of GluN2A slows the decay of the NMDA receptor component of the glutamatergic EPSC [one-way ANOVA, *F*(2,84) = 14.46, *P* < 0.0001). Mean values for each genotype: *Grin2a^+/+^*: 108.3 ± 38.77, *N* = 31; *Grin2a^+/−^*: 136.8 ± 43.61 ms, *N* = 36; *Grin2a^−/−^*: 174.2 ± 47.23, *N* = 20. Holm–Sidak post-tests: *Grin2a^+/+^* versus *Grin2a*^+/−^, *t* = 2.716, *P* = 0.0080; *Grin2a^+/+^* versus *Grin2a*^−/−^, *t* = 5.371, *P* < 0.0001. (**B–D**) The N numbers, indicated in parentheses, represent the number of animals from which recordings were obtained (i.e. the independent biological replicate). Filled symbols represent data from Line 1, open symbols from Line 2. Scale bar in **A**, 400 pA, 100 ms. Only male rats were used to generate datasets in **B**, Line 2: *Grin2a^+/+^*: 10 males; *Grin2a^+/−^*: 8 males; *Grin2a^−/−^*: 6 males. Only male rats were used to generate datasets in **C**, line 1: *Grin2a^+/+^*: 15 males; *Grin2a^+/−^*: 16 males; *Grin2a^−/−^*: 11 males. Line 2: *Grin2a^+/+^*: 6 males; *Grin2a^+/−^*: 12 males; *Grin2a^−/−^*: 7 males. Both male and female rats were used to generate datasets in **D**, line 1: *Grin2a^+/+^*: 20 males, 5 females; *Grin2a^+/−^*: 20 males, 6 females; *Grin2a^−/−^*: 12 males, 3 females. Line 2: *Grin2a^+/+^*: 6 males; *Grin2a^+/−^*: 10 males; *Grin2a^−/−^*: 5 males.

We next examined the properties of glutamatergic EPSCs recorded in CA1 pyramidal neurons evoked following stimulation of the Schaffer collateral/commissural pathway (Fig. 3A) and recorded in the presence of picrotoxin (50 μM). The nature of the current–voltage relationship of the NMDA receptor-mediated component of the EPSC in *Grin2a*^+/−^ and *Grin2a*^−/−^ neurons did not differ from that recorded from *Grin2a*^+/+^ neurons [Fig. 3B; one-way ANOVA, *F*(2,21) = 0.9202, *P* = 0.4139], as might be expected given the similar voltage-dependent Mg^2+^ block of NMDARs containing GluN2A and GluN2B subunits. In recordings from *Grin2a*^+/−^ and *Grin2a*^−/−^ CA1 pyramidal neurons we observed a genotype reduction in the NMDAR:AMPAR ratio of EPSCs [Fig. 3C; one-way ANOVA, *F*(2,64) = 4.214, *P* = 0.0191] with significant differences in ratios between *Grin2a*^+/+^ and *Grin2a*^+/−^ (*P* = 0.0169) and also *Grin2a*^+/+^ and *Grin2a*^−/−^ CA1 pyramidal neurons (*P* = 0.0264). The decay time-constant of the NMDA receptor-mediated EPSC is the fastest when mediated by GluN2A subunits and therefore consistent with a reduced or loss of GluN2A protein we observed an overall genotype effect on the decay time-constant [Fig. 3D; one-way ANOVA, *F*(2,84) = 14.46, *P* < 0.0001]. Given the dominant nature of GluN2A in determining the fast decay of NMDA receptor-mediated EPSCs,^24,29^ the complete loss of GluN2A led to a significant slowing of NMDA receptor-mediated EPSCs (*P* < 0.0001; *Grin2a*^+/+^ versus *Grin2a*^−/−^), which we have reported previously^24^ and is consistent with the notion that NMDA receptors will be predominantly comprised of GluN1 and GluN2B subunits in *Grin2a*^−/−^ CA1 neurons. We further observed a statistical difference between the decay times of EPSCs recorded from *Grin2a*^+/+^ and *Grin2a*^+/−^ neurons (*P* = 0.0080).

### Reduced dendritic complexity in *Grin2a*^−/−^ CA1 pyramidal neurons

**Figure 4:**
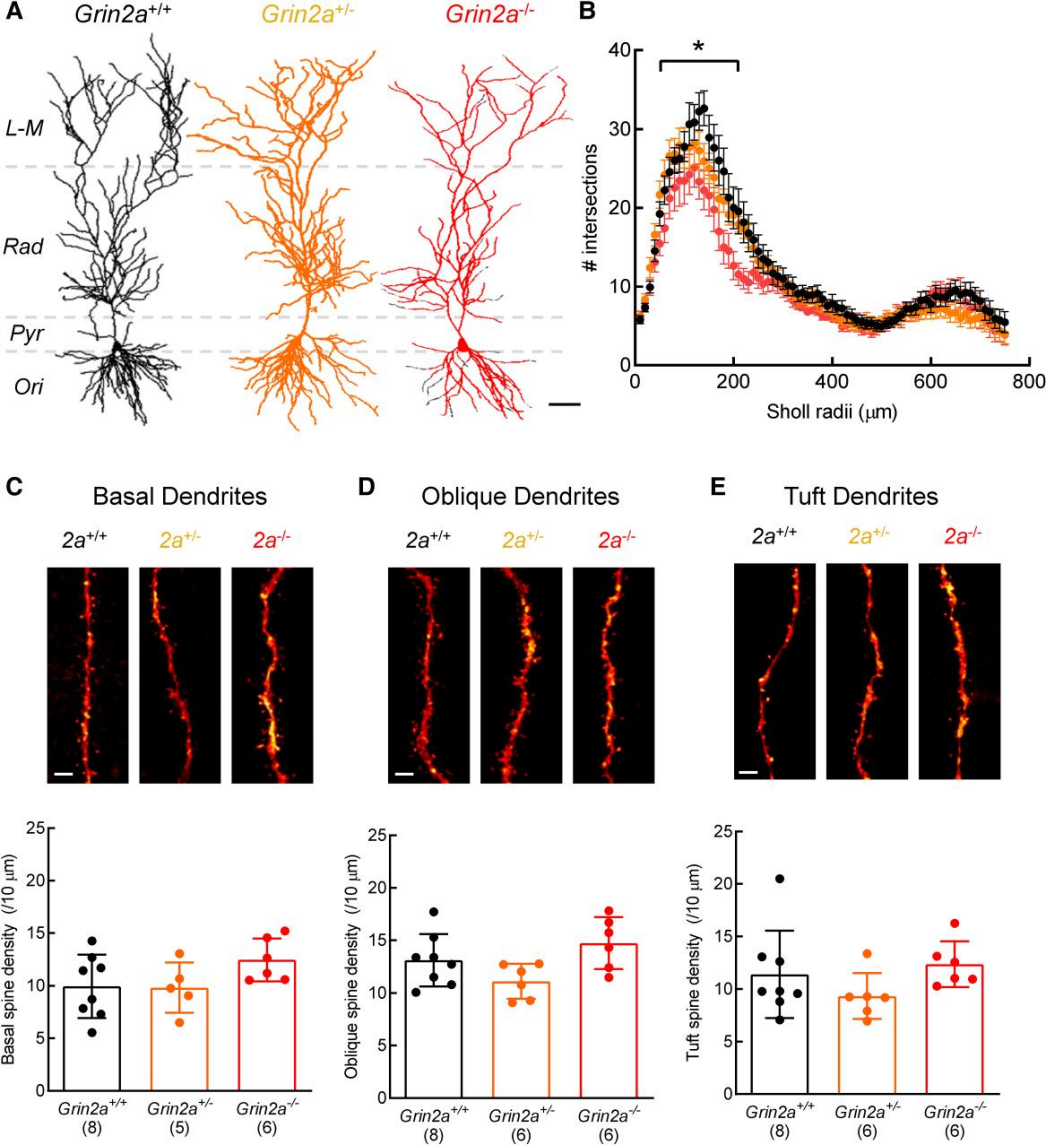
**Reduced dendritic complexity in *Grin2a*^−/−^ CA1 pyramidal neurons**. (**A**) Flattened 3-dimensional reconstructions of *Grin2a^+/+^*, *Grin2a^+/−^* and *Grin2a^−/−^* CA1 pyramidal neurons. *stratum lacunosum-moleculare, (L-M); stratum radiatum, (Rad); stratum pyramidale, (Pyr); stratum oriens, (Ori)*. (**B**) Reduction in number of Sholl intersections in *Grin2a^−/−^* compared to *Grin2a^+/+^* CA1 neurons, assessed using Sholl analysis [two-way ANOVA, *F*(22 799) = 48.42, *P* < 0.0001]. (**C**) Examples of basal dendrites from each of the three genotypes and quantification of spine density. Overall, there is no effect of genotype on basal spine density [one-way ANOVA, *F*(2,16) = 2.025, *P* = 0.1645]. Mean values for each genotype: *Grin2a^+/+^*: 9.939 ± 3.018, N = 8; *Grin2a^+/−^*: 9.808 ± 2.386, *N* = 5; *Grin2a^−/−^*: 12.46 ± 2.043, *N* = 6. **D**, as in **C**, but for oblique dendrites where an interaction of genotype with spine density is suggested [one-way ANOVA, *F*(2,17) = 3.855, *P* = 0.0416]; however, pairwise *t*-tests do not show statistical differences. Mean values: *Grin2a^+/+^*: 13.11 ± 2.495, *N* = 8; *Grin2a^+/−^*: 11.11 ± 1.659, *N* = 6; *Grin2a^−/−^*: 14.75 ± 2.471, *N* = 6. Holm–Sidak post-tests: *Grin2a^+/+^* versus *Grin2a*^+/−^, *t* = 1.633, *P* = 0.2269; *Grin2a^+/+^* versus *Grin2a*^−/−^, *t* = 1.329, *P* = 0.2269. **E**, as in **C**, but for tuft dendrites. Overall, there is no effect of genotype on tuft spine density [one-way ANOVA, *F*(2,17) = 1.464, *P* = 0.259]. Mean values for each genotype: *Grin2a^+/+^*: 11.4 ± 4.156, *N* = 8; *Grin2a^+/−^*: 9.33 ± 2.182, *N* = 6; *Grin2a^−/−^*: 12.36 ± 2.19, *N* = 6. (**C–E**) The N numbers, indicated in parentheses, represent the number of animals from which reconstructions of cells (2-4 per animal) were obtained (i.e. the independent biological replicate). Scale bars in **A**, 100 μm; **C–E**, 5 μm. Only male rats from Line 1 were used to generate datasets in **B–E.**

Activation of NMDA receptors is considered essential for the appropriate maturation of glutamatergic synapses^30^ and the developmental upregulation of GluN2A coincides with a critical period of synaptogenesis in the hippocampus. Indeed, a number of studies have established a role for NMDA receptors in dendrite development and have demonstrated that blocking them pharmacologically impairs dendritic growth and branch density.^31-33^ Furthermore, dentate granule cells show reduced dendritic complexity in GluN2A-lacking adult mice.^19^ Whether similar observations are present in the GluN2A-lacking rat remains unknown but would be important to confirm as a validation of this earlier finding. A subset of neurons recorded for electrophysiological analysis were filled with biocytin (0.2% w/v) and stained using streptavidin-conjugated fluorophore. Three-dimensional reconstructions were then made from confocal images of stained neurons to investigate the effect of alteration in subunit composition of NMDARs on dendritic growth and branch complexity (Fig. 4A). Dendritic branch complexity, assessed by Sholl analysis, was found to be reduced in *Grin2a*^−/−^ rats [Fig. 4B; two-way ANOVA, *F*(22 799) = 48.42, *P* < 0.0001], in a manner consistent with previous reports (Kannangara *et al*., 2014). Next, we examined whether dendritic morphological changes were also associated with alterations in dendritic spine density, as spines are the main site of excitatory input to CA1 pyramidal neurons. Spine densities on dendritic segments, however, within *stratum radiatum*, *stratum oriens* and *stratum lacunosum moleculare* of CA1 region were not significantly different between genotypes (Fig. 4C–E). Although there was an interaction of genotype with spine density in oblique dendrites [one-way ANOVA, *F*(2,17) = 3.855, *P* = 0.0416] *post hoc* pairwise *t*-tests do not show statistical differences. These data suggest that the absence of GluN2A subunits restricts dendritic arborization, but not the proliferation of dendritic spines,^29^ which together would be expected to lead to a numerical reduction in the number of synapses on CA1 pyramidal neurons.

### Reduced miniature EPSC frequencies in *Grin2a*^+/−^ and *Grin2a*^−/−^ CA1 pyramidal neurons

**Figure 5:**
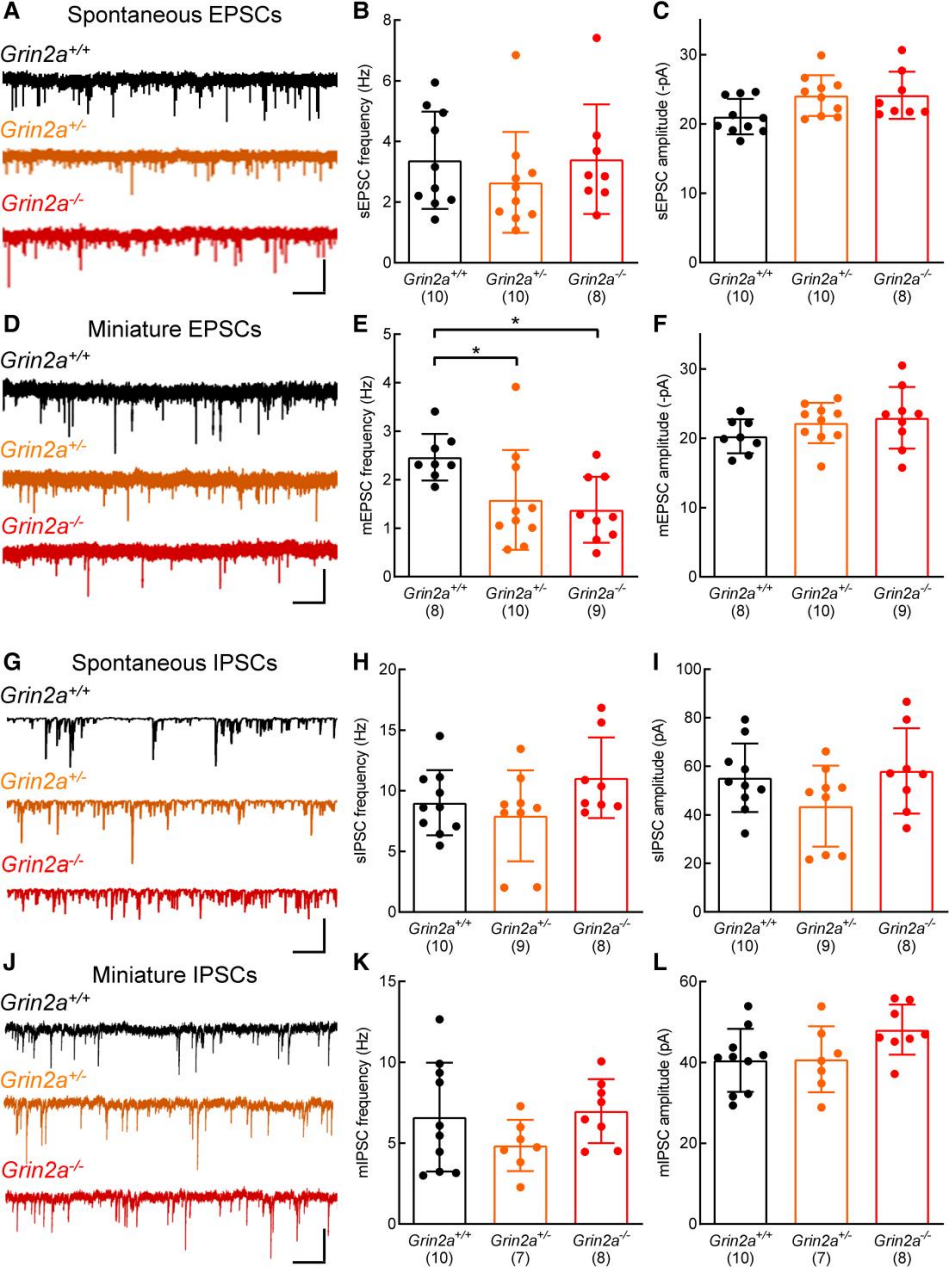
**Altered GluN2A levels lead to a reduction in miniature EPSC frequencies in CA1 pyramidal neurons.** (**A**) Example traces of spontaneous EPSCs recorded from *Grin2a^+/+^*, *Grin2a^+/−^* and *Grin2a^−/−^* CA1 pyramidal neurons voltage-clamped at −70 mV. **B**, quantification of spontaneous EPSC (sEPSC) frequencies in *Grin2a^+/+^*, *Grin2a^+/−^* and *Grin2a^−/−^* CA1 pyramidal neurons. Altered GluN2A levels do not affect event frequencies (one-way ANOVA, F(2,25) = 0.6279, *P* = 0.5419). Mean values for each genotype: *Grin2a^+/+^*: 3.378 ± 1.603 Hz, N = 10; *Grin2a^+/−^*: 2.65 ± 1.662 Hz, N = 10; *Grin2a^−/−^*: 3.415 ± 1.812 Hz, N = 8. **C**, quantification of spontaneous EPSC amplitudes in *Grin2a^+/+^*, *Grin2a^+/−^* and *Grin2a^−/−^* CA1 pyramidal neurons. Altered GluN2A levels indicate a genotype effect (one-way ANOVA, F(2,25) = 3.459, *P* = 0.0472) however pairwise t-tests do not show statistical differences. Mean values: *Grin2a^+/+^*: 21.05 ± 2.554, N = 10; *Grin2a^+/−^*: 24.09 ± 2.934, N = 10; *Grin2a^−/−^*: 24.14 ± 3.398, N = 8. Holm-Sidak post-tests: *Grin2a^+/+^* versus *Grin2a*^+/−^, t = 2.304, *P* = 0.0869; *Grin2a^+/+^* versus *Grin2a*^−/−^, t = 2.206, *P* = 0.0869. **D**, example traces of miniature EPSCs recorded from *Grin2a^+/+^*, *Grin2a^+/−^* and *Grin2a^−/−^* CA1 pyramidal neurons voltage-clamped at −70 mV. **E**, quantification of miniature EPSC (mEPSC) frequencies in *Grin2a^+/+^*, *Grin2a^+/−^* and *Grin2a^−/−^* CA1 pyramidal neurons. Altered GluN2A levels cause a reduction in event frequency (one-way ANOVA, F(2,24) = 4.499, *P* = 0.0219). F(2,24) = 4.499, *P* = 0.0219, *Grin2a^+/+^* : 2.465 ± 0.04789 Hz, N = 8; *Grin2a^+/−^* : 1.585 ± 1.029 Hz, N = 10; *Grin2a^−/−^* : 1.381 ± 0.6827 Hz, N = 9). Holm-Sidak post-tests: *Grin2a^+/+^* versus *Grin2a*^+/−^, t = 2.36, *P* = 0.0268; *Grin2a^+/+^* versus *Grin2a*^−/−^, t = 2.836, *P* = 0.0182. **F**, quantification of miniature EPSC amplitudes in *Grin2a^+/+^*, *Grin2a^+/−^* and *Grin2a^−/−^* CA1 pyramidal neurons. Altered GluN2A levels do not affect event amplitudes (one-way ANOVA, F(2,24) = 1.387, *P* = 0.2691). Mean values: *Grin2a^+/+^* : 20.28 ± 2.464 pA, N = 8; *Grin2a^+/−^* : 22.21 ± 2.899 pA, N = 10; *Grin2a^−/−^* : 22.96 ± 4.446 pA, N = 9). **G**, example traces of spontaneous IPSCs recorded from *Grin2a^+/+^*, *Grin2a^+/−^* and *Grin2a^−/−^* CA1 pyramidal neurons voltage-clamped at −70 mV. **H**, quantification of spontaneous IPSC (sIPSC) frequencies in *Grin2a^+/+^*, *Grin2a^+/−^* and *Grin2a^−/−^* CA1 pyramidal neurons. Altered GluN2A levels do not affect event frequencies (one-way ANOVA, F(2,24) = 1.9964, *P* = 0.1578). Mean values for each genotype: *Grin2a^+/+^*: 9.01 ± 2.69 Hz, N = 10; *Grin2a^+/−^*: 7.93 ± 3.75 Hz, N = 9; *Grin2a^−/−^*: 11.06 ± 3.33 Hz, N = 8. **I**, quantification of spontaneous IPSC amplitudes in *Grin2a^+/+^*, *Grin2a^+/−^* and *Grin2a^−/−^* CA1 pyramidal neurons. Altered GluN2A levels do not affect event amplitudes (one-way ANOVA, F(2,24) = 1.919, *P* = 0.1686). Mean values for each genotype: *Grin2a^+/+^*: 55.27 ± 14.11 pA, N = 10; *Grin2a^+/−^*: 43.59 ± 16.68 pA, N = 9; *Grin2a^−/−^*: 58.12 ± 17.60 pA, N = 8. **J**, example traces of miniature IPSCs recorded from *Grin2a^+/+^*, *Grin2a^+/−^* and *Grin2a^−/−^* CA1 pyramidal neurons voltage-clamped at −70 mV. **K**, quantification of miniature IPSC (mIPSC) frequencies in *Grin2a^+/+^*, *Grin2a^+/−^* and *Grin2a^−/−^* CA1 pyramidal neurons. Altered GluN2A levels do not affect event frequencies (one-way ANOVA, F(2,22) = 1.5606, *P* = 0.2324). Mean values for each genotype: *Grin2a^+/+^*: 6.61 ± 3.37 Hz, N = 10; *Grin2a^+/−^*: 4.85 ± 1.59 Hz, N = 7; *Grin2a^−/−^*: 6.98 ± 1.98 Hz, N = 8. **L**, quantification of miniature IPSC amplitudes in *Grin2a^+/+^*, *Grin2a^+/−^* and *Grin2a^−/−^* CA1 pyramidal neurons. Altered GluN2A levels do not affect event amplitudes (one-way ANOVA, F(2,22) = 2.4876, *P* = 0.1062). Mean values for each genotype: *Grin2a^+/+^*: 40.50 ± 7.78 pA, N = 10; *Grin2a^+/−^*: 40.77 ± 8.16 pA, N = 8; *Grin2a^−/−^*: 48.09 ± 6.20 pA, N = 8. For Panels **B**, **C**, **E**, **F**, **H**, **I**, **K** and **L** the N numbers, indicated in parentheses, represent the number of animals from which recordings were obtained (i.e. the independent biological replicate). Scale bars in **A**, 40 pA, 400 ms; **D**, 25 pA, 400 ms; **G**, 300 pA, 1 s; **J**, 50 pA, 600 ms. Only male rats from Line 1 were used to generate datasets in **B**, **C**, **E**, **F**, **H**, **I**, **K** and **L**.

We directly assessed synaptic activity in *Grin2a*^+/+^, *Grin2a*^+/−^ and *Grin2a*^−/−^ CA1 neurons by recording spontaneous (Fig. 5A) and miniature (Fig. 5D) ESPCs and spontaneous (Fig. 5G) and miniature (Fig. 5J) IPSCs. For spontaneous EPSCs reduced levels or loss of GluN2A did not affect their frequency [Fig. 5B; one-way ANOVA, *F*(2,25) = 0.6279, *P* = 0.5419] and while there is a suggestion of an interaction of genotype and spontaneous EPSC amplitude [Fig. 5C; one-way ANOVA, *F*(2,25) = 3.459, *P* = 0.0472], *post hoc* t-tests did not reveal any significant differences in pairwise comparisons. In the case of miniature EPSC frequencies, we observed a significant reduction in event frequencies in *Grin2a*^+/−^ and *Grin2a*^−/−^ CA1 neurons compared to recordings made from *Grin2a*^+/+^ neurons [Fig. 5E; one-way ANOVA, *F*(2,24) = 4.499, *P* = 0.0219]. Reduced or loss of GluN2A did not significantly change quantal size (Fig. 5F; one-way ANOVA, *F*(2,24) = 1.387, *P* = 0.2691). Furthermore, we uncovered no impact of GluN2A depletion or loss on spontaneous IPSC frequency [Fig. 5H; one-way ANOVA, *F*(2,24) = 1.9965, *P* = 0.1578] or amplitude [Fig. 5I; one-way ANOVA, *F*(2,24) = 1.919, *P* = 0.1686]. Miniature IPSCs were similarly unaffected, whereby both frequency [Fig. 5K; one-way ANOVA, *F*(2,22) = 1.5606, *P* = 0.2324] and amplitude [Fig. 5L; one-way ANOVA, *F*(2,22) = 2.4876, *P* = 0.1062] remain unaltered.

### No alterations in EPSP summation and synaptically-evoked action potential firing with reduced GluN2A levels

**Figure 6:**
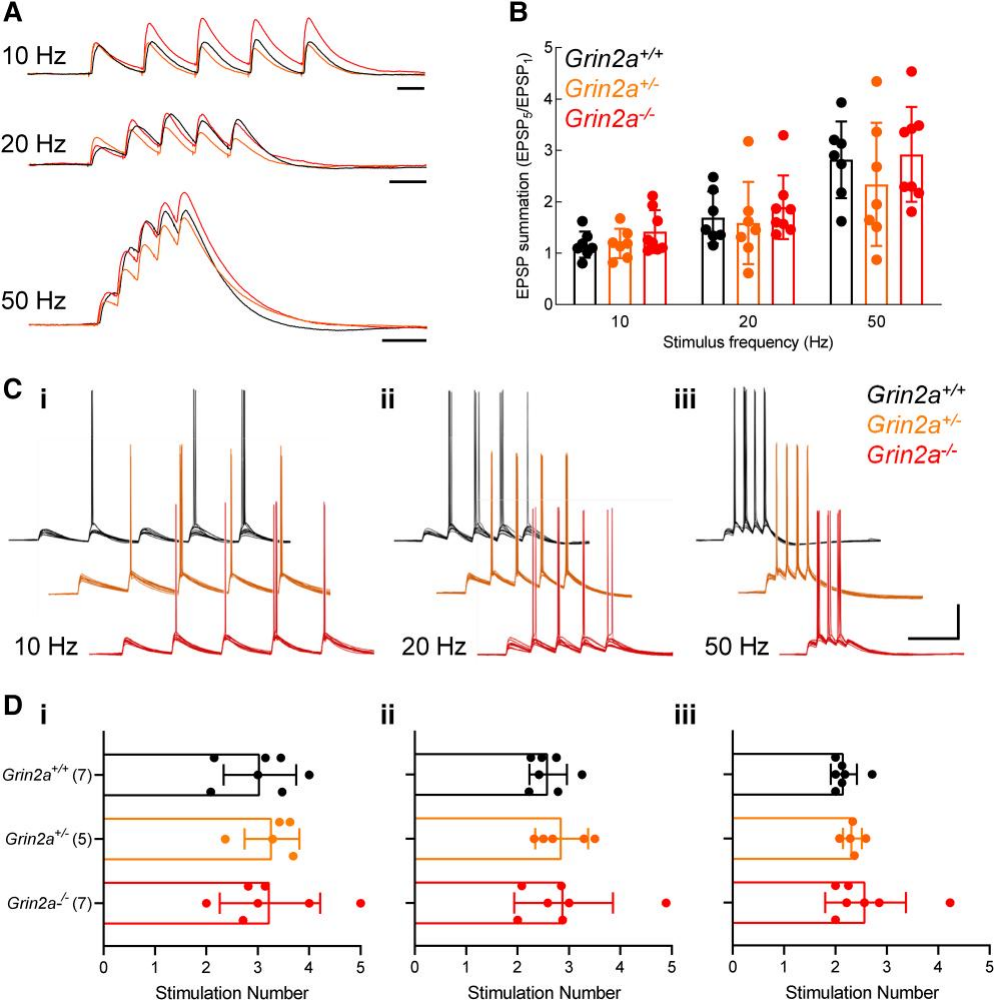
**No alterations in synaptic integration with reduced GluN2A levels**. (**A**) Example traces of five subthreshold summating EPSPs recorded from *Grin2a^+/+^*, *Grin2a^+/−^* and *Grin2a^−/−^* CA1 pyramidal neurons, at 10, 20 and 50 Hz stimulation frequencies. (**B**) Quantification of EPSP summation, expressed as the final EPSP peak amplitude normalized to the first EPSP peak in *Grin2a^+/+^*, *Grin2a^+/−^* and *Grin2a^−/−^* CA1 pyramidal neurons. Altered GluN2A levels do not affect temporal summation [two-way ANOVA, effect of genotype: *F*(2,19) = 0.7309, *P* = 0.4945; effect of stimulation frequency: *F*(2,38) = 57.50, *P* < 0.0001; interaction: *F*(4,38) = 0.6200, *P* = 0.6510]. Mean values for each genotype at 10 Hz stimulation frequency: *Grin2a^+/+^*: 1.164 ± 0.257 Hz, N = 7; *Grin2a^+/−^*: 1.188 ± 0.286 Hz, *N* = 7; *Grin2a^−/−^*: 1.420 ± 0.417 Hz, *N* = 8. Mean values for each genotype at 20 Hz stimulation frequency: *Grin2a^+/+^*: 1.693 ± 0.504 Hz, *N* = 7; *Grin2a^+/−^*: 1.586 ± 0.801 Hz, *N* = 7; *Grin2a^−/−^*: 1.892 ± 0.619 Hz, *N* = 8. Mean values for each genotype at 50 Hz stimulation frequency: *Grin2a^+/+^*: 2.816 ± 0.747 Hz, *N* = 7; *Grin2a^+/−^*: 2.338 ± 1.200 Hz, *N* = 7; *Grin2a^−/−^*: 2.920 ± 0.923 Hz, *N* = 8. (**C**) Example traces of five suprathreshold summating EPSPs recorded from *Grin2a^+/+^*, *Grin2a^+/−^* and *Grin2a^−/−^* CA1 pyramidal neurons, at 10 (i), 20 (ii) and 50 Hz (iii) stimulation frequencies. (**D**) Quantification of EPSP summation-induced spiking in *Grin2a^+/+^*, *Grin2a^+/−^* and *Grin2a^−/−^* CA1 pyramidal neurons, expressed as the mean stimulation number in which the first action potential was initiated, during 10 (i), 20 (ii) and 50 Hz (iii) stimulations. Altered GluN2A levels do not affect spike initiation [two-way ANOVA, effect of genotype: *F*(2,16) = 0.5743, *P* = 0.5743; effect of stimulation frequency: *F*(2,32) = 14.40, *P* < 0.0001; interaction: *F*(4,32) = 0.1848, *P* = 0.9446]. Mean values for each genotype at 10 Hz stimulation frequency: *Grin2a^+/+^*: 3.044 ± 0.707, *N* = 7; *Grin2a^+/−^*: 3.277 ± 0.534, *N* = 5; *Grin2a^−/−^*: 3.238 ± 0.978, *N* = 7. Mean values for each genotype at 20 Hz stimulation frequency: *Grin2a^+/+^*: 2.594 ± 0.363, *N* = 7; *Grin2a^+/−^*: 2.859 ± 0.514, *N* = 5; *Grin2a^−/−^*: 2.697 ± 0.962, *N* = 7. Mean values for each genotype at 50 Hz stimulation frequency: *Grin2a^+/+^*: 2.163 ± 0.253, *N* = 7; *Grin2a^+/−^*: 2.331 ± 0.184, *N* = 5; *Grin2a^−/−^*: 2.586 ± 0.786, *N* = 7. Scale bars in **A**, 50 ms; **C**, 25 mV, 100 ms. Only male rats from line 1 were used to generate datasets in **B** and **D**.

NMDA receptors are known to mediate temporal integration of synaptic inputs, attributed to their contributions to the slow decay phase of the EPSP. We hypothesized that the slower decay kinetics of GluN2B would, in principle, facilitate a longer decay phase to EPSPs, leading to a subsequent increase in the temporal summation. We assessed synaptic integration by measuring subthreshold EPSP summation at different frequencies of Schaffer collateral stimulation (10, 20 and 50 Hz, Fig. 6A). Summation, presented as the amplitude of the last EPSP normalized to the amplitude of the first EPSP in each train, was greater at higher frequencies [Fig. 6B; two-way ANOVA, effect of stimulation frequency: *F*(2,38) = 57.50, *P* < 0.0001]. However, overall summative capacity appears unchanged by GluN2A reduction or deletion [Fig. 6B; two-way ANOVA, effect of genotype: *F*(2,19) = 0.7309, *P* = 0.4945]. Finally, to determine whether reduced or absent GluN2A led to a change in synaptic integration leading to action potential firing we conducted a similar set of experiments but with larger amplitude initial EPSPs and determined the mean stimulus number within a train that evoked the first action potential (Fig. 6C), again using the same inter-stimulus intervals of 10, 20 and 50 Hz. While we observed a frequency-dependent decrease in the stimulus number required to evoke an action potential [Fig. 6D; two-way ANOVA, effect of stimulation frequency: *F*(2,32) = 14.40, *P* < 0.0001], this was not dependent on genotype [Fig. 6D; two-way ANOVA, effect of genotype: *F*(2,16) = 0.5743, *P* = 0.5743]. Thus, while slower EPSCs are observed when GluN2A levels are reduced or absent, EPSPs summation and synaptically-driven action potential firing is similar in *Grin2a*^+/+^, *Grin2a*^+/−^ and *Grin2a*^−/−^ CA1 pyramidal cells.

## Discussion

In the current study, we characterize the cellular and synaptic structure and function of CA1 pyramidal neurons of the hippocampus in rats lacking functional copies of one or both alleles of *Grin2a*. We show in two rat lines that either partial or full knock-out of *Grin2a* leads to selective loss of GluN2A subunits, which has the effect of reducing the amplitudes and slowing the kinetics of NMDA receptor-mediated synaptic currents. These data indicate that loss or reduction in GluN2A lead to changes in NMDA receptor-mediated synaptic currents and alteration in basic synaptic properties, which would be predicted to influence circuit-level integration.

### Altered synaptic NMDAR currents

The primary function of NMDA receptors is to contribute to ionotropic synaptic neurotransmission. The presence of the GluN2A subunit speeds up NMDA receptor kinetics; thus its absence or reduction will shift the balance of synaptic receptors to favour those containing the kinetically slower GluN2B subunit. We observed a significant slowing of the NMDA receptor component of EPSCs in *Grin2a^+/−^* and *Grin2a*^−/−^ rats, in agreement with previous studies knocking out *Grin2a* in mouse acute hippocampal slices.^15,29,34^ However, in both genotypes, we observed a net reduction of NMDA receptors overall, demonstrated by reduced NMDA/AMPA ratios. As expected, the overall voltage-dependence of pharmacologically isolated NMDA receptor-mediated EPSCs in all genotypes were unchanged, consistent with the near-identical Mg^2+^ blockade of GluN2A and GluN2B containing receptors.^35,36^ We did not see a previously hypothesized significant decrease in GluN1 protein levels or a compensatory increase in GluN2B subunits, consistent with previous findings from our lab in neuronal cultures from Line 1.^14^

### Absence of GluN2A leads to altered CA1 pyramidal cell structure

We observed a consistent reduction in the complexity of the dendrites of *Grin2a*^+/−^ CA1 pyramidal cells, which was most prominent in the proximal dendritic domain. Alongside a reduction in overall complexity, there was a tendency towards shorter dendritic arborization, but this did not reach statistical significance. Moreover, membrane capacitance, which is a proxy for somatodendritic surface area, did not show any genotype dependence. Although synaptic density, as measured from imaged neurons, was not reduced but miniature EPSC frequency was—in both *Grin2a^+/−^* and *Grin2a^−/−^* CA1 neurons—this might suggest that the total synapse number of a given neuron may be lower when GluN2A is reduced or absent. A simple arithmetic comparison of total dendrite length with average spine density suggests that CA1 principal cells in *Grin2a*^+/+^ rats possess an estimated average of 14 528 ± 884 spines, with *Grin2a^+/−^* and *Grin2a^−/−^* having 13 191 ± 964 and 12 571 ± 458, respectively (a 9% and 13% reduction). We accept that, while not statistically significant, this may reflect an overall tendency towards reduced excitatory synapse numbers. Such an effect would be consistent with the finding of reduced dendritic complexity and reduction in length in dentate gyrus inner granular zone neurons in *Grin2a^−/−^* mice.^19^ The more subtle and non-significant effect we observe here may result from a degree of compensation for GluN2A absence or its reduction throughout development. Determining how neuronal structure and synapse density change at spinogenesis (<10 days postnatally) may reveal more precise mechanisms of neuronal dysfunction caused by reduced expression of *Grin2a*. Previous studies investigating the role of GluN2A, postnatally, in shaping dendritic morphology and miniature EPSC properties have yielded contrasting results to those we report here. In hippocampal neurons, reducing GluN2A levels resulted in increased miniature EPSC amplitudes but no change in their frequencies^29^ whereas overexpression studies of GluN2A in organotypic slice cultures led to decreased miniature EPSC frequencies, no change in their amplitude but an overall decrease in the number of dendritic spines.^37^

### Functional ramifications for loss of GluN2A

We have shown here that loss of GluN2A has modest effects on hippocampal CA1 neuronal function. However, in humans, loss-of-function variants of *GRIN2A* are associated with an increased risk for epilepsy, intellectual disability and schizophrenia.^12,14^ These disruptions in excitability and cognition are not explained by the data we present concerning CA1 pyramidal neurons. Indeed, our data on basal activity of CA1 neurons does not indicate changes to cell-autonomous functions linked to widespread epileptogenesis. While slowing of EPSC decays and reduced NMDA:AMPA receptor EPSC amplitude ratios were evident in recordings obtained from *Grin2a^+/−^* and *Grin2a*^−/−^ pyramidal neurons, our data highlight the difficulty in predicting how such changes seen in voltage-clamp recordings manifest as changes in EPSPs. In current-clamp recordings, EPSP profiles did not change to an extent sufficient to influence either summation of sub-threshold EPSPs or summation of supra-threshold EPSPs to initiate action potential firing. Furthermore, it is possible that a subsequent developmental stage may have yielded different results (in mice, the largest transcriptomic effects are seen at 12 weeks^22^), and the relatively narrow developmental window studied is a limitation of our findings. We also did not observe any spontaneous seizures in these rat lines during general husbandry and while it is possible that subtle epileptiform activity, such as absence seizures, were missed such activity would require *in vivo* EEG recordings, which were beyond the remit of the current study. In agreement with our data, longitudinal assay of seizure activity in *Grin2a*-null mice did not note any observable seizures but did find transient epileptiform activity.^21,38^

This raises an interesting conundrum—what leads to epilepsy in humans with reduced GluN2A? One possibility is that, beyond the isolated activity of principal neurons, seizure activity results from cumulative changes within local networks, in particular, interactions with inhibitory neurons and non-neuronal cells and the balance of excitation to inhibition. Indeed, using the same model (Line 1) we showed that inhibitory interneurons in the CA1 region contain GluN2A to varying degrees.^24^ This is especially true of somatostatin (SST)-containing interneurons, which possessed the highest contingent of GluN2A, relative to other classes of interneuron. Indeed, SST interneurons in the rodent dentate gyrus are known to synchronise activity during seizure activity *in vitro*^39^ and are especially vulnerable in models of temporal lobe epilepsy.^40^ Given that SST interneurons are the archetypal feedback inhibitory element in cortical circuits, their potentially reduced recruitment to repetitive circuit activity following loss of *Grin2a* may lead to the GABA-dependent neuronal synchrony that precedes seizure onset.^41^ Supporting this idea is the observation that there is a transient delay in the electrophysiological signature of hippocampal parvalbumin interneurons in *Grin2a^+/−^* and *Grin2a^−/−^* mice^15^ and this study noted that individuals with *GRIN2A* null mutations showed a later age of onset of seizures compared to those individuals carrying missense *GRIN2A* mutations. Furthermore, there is a reported higher incidence of seizure offset in patients with *GRIN2A* null mutations.^15^

In summary, we show in two rat lines that absence or haploinsufficiency of *Grin2a* alters the kinetics of NMDAR-mediated synaptic events and dendritic structure of CA1 neurons. We show that core features of pyramidal neuronal function, including intrinsic excitability, synaptic function, and synaptic integration are largely unaffected by either heterozygous or homozygous deletion of *Grin2a*. It is possible that the changes in synaptic properties seen in *Grin2a* haploinsufficiency lead to more subtle effects on higher-level circuit dynamics and computation which may manifest as disorders of cognition and excitability in humans.

## Supplementary Material

fcaf124_Supplementary_Data

## Data Availability

The data that support the findings of this study are available from the corresponding author upon reasonable request.
