# Supplementary figures and images for: Absence of GluN2A in hippocampal CA1 neurons leads to altered dendritic structure and reduced frequency of miniature excitatory synaptic events
Source: Brain Commun. 2025 Mar 26;7(2):fcaf124. doi: 10.1093/braincomms/fcaf124 (PMC11986202; doi:10.1093/braincomms/fcaf124)

## Supplemental Figure 2: Full blots for Line 2 in Figure 1.

○ *Grin2a*<sup>+/+</sup>  
○ *Grin2a*<sup>+/-</sup>  
○ *Grin2a*<sup>-/-</sup>

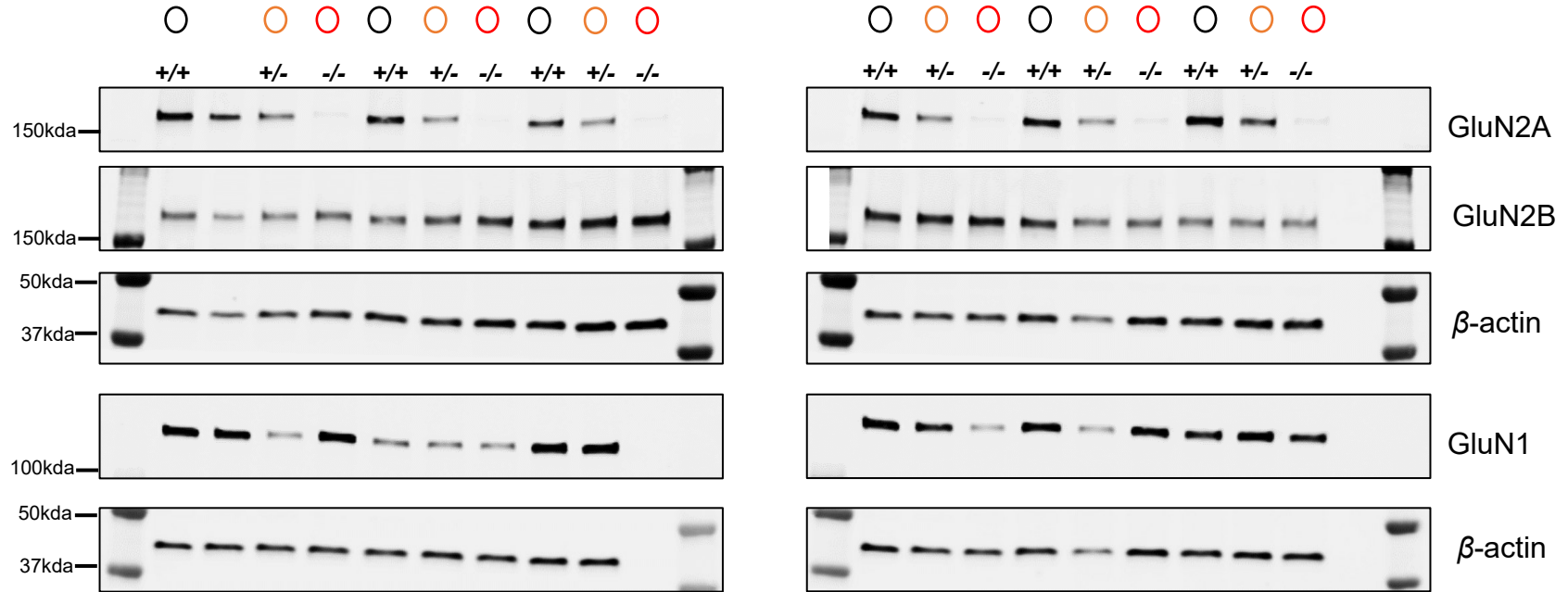

Supplement: fcaf124_Supplementary_Data [file fcaf124_supplementary_data.pdf]
